# Diseases of the Rectum

**Published:** 1887-12

**Authors:** 


*Diseases of the Rectum.* By ALFRED COOPER, F.R.C.S. Pp. 180. London: H. K. LEWIS. 1887.

There is little in this book which is either new or untrue. At least half a dozen works on general surgery in the English language give better practical accounts of Diseases of the Rectum than this one. The language is rarely elegant, and often slovenly; and we have marked many sentences as being grammatically deficient. There is a want of knowledge of the work of others which is by no means made up for by the "ample opportunities for investigation and treatment" which the author has enjoyed "both in private practice and at St. Mark's Hospital." It is an undeniable fact that many a midwife has had "opportunities for investigation and treatment" more ample than the most distinguished accoucheur living; but that need not justify the woman in writing a book on Midwifery.

